# Rhizosphere microbiome differentiation and soil environmental drivers in two *Monotropastrum* species

**DOI:** 10.71150/jm.2602009

**Published:** 2026-07-31

**Authors:** Qian Liu, Xiaorong Chen, Xi Liu, Lingjuan Liu, Cuiting Chen, Lingling Li, Weiqing Liang, Pan Xu, Jinbao Pu

**Affiliations:** 1 School of Pharmacy, Zhejiang Chinese Medical University, Hangzhou 310053, P. R. China; 2 Qingyuan Preserve Center of Qianjiangyuan-Baishanzu National Park, Lishui 323800, P. R. China; 3 The Management Center of Wuyanling National Nature Reserve of Zhejiang, Wenzhou 325500, P. R. China; 4 Longquan Preserve Center of Qianjiangyuan-Baishanzu National Park, Lishui 323700, P. R. China; 5 Zhejiang Academy of Traditional Chinese Medicine, Hangzhou 311308, P. R. China; 6 Tongde Hospital of Zhejiang Province, Hangzhou 311308, P. R. China

**Keywords:** *Monotropastrum humile*, *M. humile* var. *glaberrima*, rhizosphere microbial community, high-throughput sequencing, soil physicochemical properties

## Abstract

This study compared the rhizosphere microbial communities of two closely related *Monotropastrum* species (*M. humile*, Mh; and *M. humile* var. *glaberrima*, Mhg) and identified key soil factors associated with their assembly. Bacterial and fungal communities were profiled by Illumina high-throughput sequencing, and soil physicochemical properties were assessed across multiple sites in Zhejiang Province, China. The bacterial communities of both species were dominated by Proteobacteria and Acidobacteriota at the phylum level, while the dominant fungal groups belonged to Ascomycota and Basidiomycota. The two plants shared several dominant bacterial genera, including *Serratia*, *Burkholderia-Caballeronia-Paraburkholderia*, and *Bradyrhizobium*, as well as common dominant fungal genera such as *Saitozyma* and *Podila*. Despite these similarities, species-specific enrichment patterns were observed. The rhizosphere of Mhg contained higher abundances of *Acidothermus* and *Lactarius*, whereas Mh preferentially enriched *Cedecea*, *Klebsiella*, and *Russula*. Bacterial communities were shaped by pH, soil organic matter (SOM), available potassium (AK), and available phosphorus (AP), whereas fungal communities were primarily influenced by pH, alkali-hydrolyzable nitrogen (AN), and SOM (*p* < 0.05). These results suggest that both host identity and soil properties contribute to rhizosphere microbial assembly, with clear host-associated differentiation in microbial communities. Notably, the identified host-associated microbial taxa, particularly key mycorrhizal fungi, may serve as potential microbial inoculants, providing new opportunities for the conservation and cultivation of mycoheterotrophic plants.

## Introduction

*Monotropastrum humile* (Mh) and its glabrous variant *M. humile* var. *glaberrima* (Mhg) are achlorophyllous, mycoheterotrophic plants belonging to the genus *Monotropastrum*. These taxa exhibit a restricted distribution in Asia (Hara, 1969). Their flowering displays are visually striking (Tsukaya et al., 2008), and both have been utilized in traditional Chinese medicine for the treatment of cough, indicating notable ornamental and potential medicinal value. However, due to their highly specific growth requirements, limited reproductive capacity, and difficulties in artificial cultivation, naturally populations of these species are rare. Therefore, understanding their growth and development patterns and exploring potential strategies for their conservation and propagation are of considerable ecological and practical importance.

Both Mh and its variant Mhg completely lack chlorophyll and the capacity for photosynthesis (Leake, 1994), relying entirely on mycoheterotrophy. This nutrition mode is mediated by a mycorrhizal symbiosis, a structure formed between their root systems and specific soil fungi, which absorbs nutrients from the soil to provide the plants with their complete nutritional supply (Bidartondo and Bruns, 2005; Leake et al., 2004; Selosse et al., 2006). Previous studies have revealed that these *Monotropastrum* species predominantly forms specific symbiotic associations with ectomycorrhizal fungi of the genus *Russula* and *Lactarius* (Liu et al., 2024; Matsuda et al., 2011). While the mycorrhizal fungal compositions of both plant species have been partially characterized, the broader rhizosphere microbial communities—encompassing both bacterial and fungal assemblages—as well as the edaphic factors shaping these communities, remain largely unexplored.

Rhizosphere microorganisms play critical roles in plant growth and soil nutrient cycling (Cao et al., 2023; Igiehon and Babalola, 2018; Wang et al., 2020). These microbial communities are also known to improve plant resilience to various abiotic stresses and certain rhizosphere fungi form mycorrhizal associations with roots, significantly improving plant nutrient uptake efficiency (Duan et al., 2024; Jiang et al., 2023; Khoso et al., 2024; Xu et al., 2025; Yuan et al., 2018). Saprotrophic microorganisms in the plant rhizosphere can also enhance the colonization rate of mycorrhizal fungi on plant roots, thereby improving rhizosphere nutrient supply to the host plant (Cao et al., 2022). In addition, recent works have highlighted that plant-associated microbiomes are strongly influenced by environmental gradients, plant functional traits, and genetic variation. For instance, flooding regimes have been shown to restructure bacterial, fungal, and archaeal communities in riparian wetlands (Qiqige et al., 2025), while vegetation type markedly shapes soil bacterial assemblages on lava plateaus (Zhang et al., 2025), variations in lotus root cultivars have also been linked to distinct rhizosphere microbial profiles (Li et al., 2025). These findings collectively emphasize that both host identity and environmental conditions jointly govern the composition and function of plant-associated microbiomes.

Yet, no study has systematically compared rhizosphere microbial communities between closely related *Monotropastrum* taxa or explored how soil properties mediate their assembly. Furthermore, it remains unclear whether mycoheterotrophic plants that co-occur in similar habitats harbor similar or distinct rhizosphere microbial communities. Sympatric Mh and Mhg provide a natural comparative system to examine whether host-associated differences in microbial community composition can be detected under comparable environmental conditions. Such comparisons are important for disentangling host-related effects from environmental influences in shaping rhizosphere microbiomes.

To address this gap, the present study employs Illumina sequencing to analyze the composition and diversity of microbial communities in the rhizosphere soil of Mh and Mhg across different sites in Zhejiang Province, China. This research aims to: (1) elucidate the composition, diversity, and distribution characteristics of both bacterial and fungal communities in the rhizosphere of the two species of *Monotropastrum*; (2) investigate the influence of soil physicochemical properties on the structure of the dominant soil microbial communities and identify the key environmental drivers shaping the rhizosphere soil microbiomes of these two plants. The findings of this study are expected to provide a research foundation for exploring potential plant growth-promoting microbial resources associated with these two species.

## Materials and Methods

### Study site description

The rhizosphere soil of two *Monotropastrum* species were collected from their natural habitats in Zhejiang Province, China, in April 2024. Rhizosphere soil of the two species were sampled from three distinct distribution areas each. Specifically, soils from Jingning (JN), Taishun (TS), and Yongkang (YK) were associated with Mh, while those from Baizhangling (BZL), Longquan (LQ), and Qingyuan (QY) were associated with Mhg. The sampling sites, characterized by a subtropical monsoon climate with moderate temperatures, distinct seasons, abundant sunlight, and high rainfall, exhibited an annual mean temperature of 15–18°C and mean annual precipitation of 1,100–2,000 mm. Detailed site information is provided in Table S1. The sites were distributed across four distinct forest vegetation types: mixed coniferous and broad-leaved forests, coniferous forests, deciduous broad-leaved forests, and evergreen broad-leaved forests.

### Soil samples collection

Prior to excavation, all sampling tools were sterilized with 75% ethanol to avoid cross-contamination. At each of the six distribution sites, healthy *Monotropastrum* plants were randomly selected. Surface debris was carefully removed, and the plants along with their mycorrhizal roots were gently excavated. Soil adhering within approximately 5 mm of the root surface was gently shaken off and collected as rhizosphere soil. To ensure sample representativeness, three sampling points were established at each site. At each site, rhizosphere soil from 10 individual plants were pooled and mixed into a single composite sample stored in a sterile plastic bag, yielding a total of 18 independent soil samples. Each composite sample was sieved through a 10-mesh sieve. One subsample was immediately stored at -80°C for bacterial and fungal DNA extraction and high-throughput sequencing, while the remaining portion was air-dried for determination of soil physicochemical properties. Representative plant samples collected in this study are shown in Fig. 1.

### Determination of soil physicochemical properties

The soil pH was determined potentiometrically at a controlled soil-to-water ratio of 1:5 (Yang et al., 2020). Soil organic matter (SOM) content was quantified by K_2_Cr_2_O_7_ oxidation under external heating at 170°C (Zhu et al., 2020), followed by titration of residual oxidant with ferrous sulfate. Soil total nitrogen (TN) was determined by Kjeldahl digestion followed by indophenol blue colorimetry, while alkali-hydrolyzable nitrogen (AN) was quantified via alkali-diffusion coupled with indophenol blue spectrophotometry (Hailu and Betemariyam, 2021). Total phosphorus (TP) was determined by the antimony-molybdate colorimetric method after sulfuric-perchloric acid digestion, while available phosphorus (AP) was quantified using the molybdenum blue colorimetric method. Available potassium (AK) content was determined by sodium tetraphenylboron turbidimetry (Wang et al., 2010).

### DNA extraction and PCR amplification

Total microbial DNA was extracted from soil samples using the FastPure Soil DNA Isolation Kit (Magnetic bead) (MJYH, China). DNA concentration and purity were assessed spectrophotometrically using a NanoDrop 2000 instrument (Thermo Scientific, USA). The hypervariable V3–V4 region of bacterial 16S rRNA genes was amplified with primers 338F (5′-ACTCCTACGGGAGGCAGCAG-3′) and 806R (5′-GGACTACHVGGGTWTCTAAT-3′), whereas the fungal ITS1 region was targeted using primers ITS1F (5′-CTTGGTCATTTA GAGGAAGTAA-3′) and ITS2R (5′-GCTGCGTTCTTCATCGATGC-3′). The PCR reaction mix contained 5×TransStart FastPfu (4 μl), 2.5 mmol/L dNTPs (2 μl), 5 μmol/L each of forward and reverse primers (0.8 μl), 0.4 μl Fast Pfu polymerase, 10 ng of template DNA, and ddH_2_O to a final volume of 20 µl. PCR amplification cycling conditions were as follows: initial denaturation at 95℃ for 3 min, followed by 27 cycles of denaturing at 95℃ for 30 s, annealing at 55℃ for 30 s and extension at 72℃ for 45 s, and single extension at 72℃ for 10 min, and end at 4℃. The PCR product was extracted from 2% agarose gel and purified using the PCR Clean-Up Kit (YuHua, China) according to manufacturer’s instructions and quantified using Qubit 4.0 (Thermo Fisher Scientific, USA).

### Illumina sequencing and data processing

Purified amplicons were pooled in equimolar amounts and paired-end sequenced on an Illumina Nextseq 2000 platform (Illumina, USA) according to the standard protocols by Majorbio Bio-Pharm Technology Co. Ltd. (China). The raw data were uploaded to the NCBI database for comparison. Paired-end raw sequencing reads were processed using Fastp (https://github.com/OpenGene/fastp, v0.19.6) for quality control and adapter trimming, followed by sequence assembly with FLASH (http://www.cbcb.umd.edu/software/flash, version 1.2.11). Effective sequences were clustered into operational taxonomic units (OTUs) at a 97% similarity threshold using UPARSE v7.1 (Edgar, 2013). Taxonomic annotation of the Operational taxonomic unit (OTUs) was performed by comparing the gene database using RDP classifier (http://rdp.cme.msu.edu/, version 2.11).

### Statistical analysis

Experimental data on physical and chemical soil properties were statistically analyzed using Excel 2016 and GraphPad Prism 10. Results are presented as the Mean ± standard deviation (SD). All samples were rarefied to an equal sequencing depth (34,994 reads per sample) prior to alpha and beta diversity analyses. Alpha-diversity indices were calculated using Mothur software (https://mothur.org/wiki/calculators/, version v.1.30.2), and intergroup differences were assessed by Kruskal Wallis rank-sum test. R version 3.3.1 was used to perform non-metric multidimensional scaling (NMDS) based on Bray-Curtis distances, to generate Venn plots, conduct PERMANOVA analysis, and evaluate Spearman correlations between microbial communities composition and soil physicochemical properties. Sample-species relationship diagrams were generated using Circos-0.67-7, and Gephi v.0.10.1 was applied for co-occurrence network visualization. The microbial groups with significant abundance differences between two *Monotropastrum* species from phylum to genus level were identified using linear discriminant analysis (LDA) effect size (LEfSe), with an LDA score> 3.5 and *p* ≤ 0.05 as thresholds for significant enrichment. Functional annotation of bacterial and fungal communities was performed using FAPROTAX (v1.2.1) and FUNGuild (http://www.funguild.org/), respectively.

## Results

### Analysis of the physicochemical properties of soil for two *Monotropastrum* species

The rhizosphere soil pH across all sampling sites ranged from 4.58 to 5.60, indicating that both *Monotropastrum* taxa occurred in acidic soil environments. Within-species comparisons showed significant site-related variation in several physicochemical properties, including alkali-hydrolyzable nitrogen (AN), total nitrogen (TN), and available potassium (AK) (*p* < 0.05; Table S2). However, no significant differences were detected in the measured rhizosphere soil physicochemical properties between Mh and Mhg (*p* > 0.05; Table S3).

### Venn diagram analysis of soil microorganisms in rhizosphere of two *Monotropastrum* species

Venn diagram analysis revealed both shared and unique microbial taxa among sampling sites and between the two *Monotropastrum* species. A total of 5,442 and 5,073 bacterial OTUs were identified in Mh and Mhg, respectively, along with 2,819 and 2,659 fungal OTUs. Within each species, a considerable proportion of OTUs were shared across sites, indicating the presence of a core microbiome, while a substantial number of unique OTUs reflected spatial variability in microbial community composition.

When comparing between the two species, 3,188 bacterial OTUs and 977 fungal OTUs were shared, suggesting a broadly similar microbial background. However, Mh exhibited slightly higher numbers of unique bacterial and fungal OTUs than Mhg, indicating species-specific differentiation in rhizosphere microbial communities (Fig. 2).

### Diversity of rhizosphere soil bacterial and fungal communities

Alpha diversity indices (Chao1, ACE, Simpson, Shannon) were computed to assess microbial community richness and diversity across sampling sites (Table 1). Among Mh samples, those from Yongkang (YK) showed the highest ACE and Chao1 indices, with a bacterial Shannon index of 5.80 ± 0.43 and fungal Shannon index of 3.99 ± 0.24, demonstrating both high richness and diversity in its microbial communities. In contrast, Mhg samples from Longquan (LQ) exhibited the highest bacterial and fungal ACE and Chao1 indices, indicating greater microbial richness in its rhizosphere soil. Comparative analysis of alpha diversity indices (Table 2) revealed no significant differences in bacterial community diversity between the two *Monotropastrum* species (*p* > 0.05). However, the fungal community in the Mh rhizosphere displayed a significantly higher Simpson index than that of Mhg (*p* < 0.05), suggesting greater fungal evenness in Mhg.

At the OTU level, non-metric multidimensional scaling (NMDS) analysis revealed bacterial communities from three Mh sampling regions clustered closely in the NMDS ordination space, whereas fungal communities exhibited distinct separation (Fig. 3A and 3B), suggesting similar bacterial community composition but divergent fungal assemblages across regions (*p* < 0.05). For Mhg, clear separation of bacterial and fungal communities across three sampling regions (Fig. 3C and 3D), indicating significant differences in microbial community composition (*p* < 0.05). Comparative NMDS analysis of two *Monotropastrum* species (Fig. 3E and 3F) showed considerable overlap in bacterial community composition between their rhizosphere, but minimal overlap for fungal communities (*p* < 0.05). These results confirmed that bacterial communities were phylogenetically conserved between the two *Monotropastrum* species, whereas fungal communities differed significantly.

To further evaluate the contributions of host plant identity and the soil properties to the assembly of rhizosphere microbial communities, a permutational multivariate analysis of variance (PERMANOVA) was performed. The results (Table 3) showed that plant species (Mh & Mhg) explained a significant proportion of the variation in rhizosphere microbial community structure (*p* < 0.05), accounting for 11.8% and 55.24% of the variation (R²) in bacterial and fungal communities, respectively. Among the seven measured soil environmental factors, soil pH (*p* < 0.05, R² = 14.41%) and available potassium (AK) content (*p* < 0.05, R² = 15.83%) had significant effects on the bacterial community. For the fungal community, significant associations were observed with soil total nitrogen (TN) (*p* < 0.05, R² = 4.63%), AK (*p* < 0.05, R² = 5.09%), and SOM (*p* < 0.05, R² = 4.68%). Notably, plant species explained a relatively large proportion of the variation in fungal communities, indicating strong host-associated differentiation. Overall, these results indicate that both host plant specificity and soil properties contribute to the structuring of rhizosphere microbial communities, with host-associated differentiation evident between the two *Monotropastrum* species.

### The composition of microbial communities in the rhizosphere soil of two *Monotropastrum* species

At the bacterial phylum level, the dominant taxa in the rhizosphere soils of both *Monotropastrum* species were Proteobacteria, Acidobacteriota and Actinobacteriota (Fig. 4A). Among these Proteobacteria was the predominant phylum, accounting for 56.76% and 62.45% of the total bacterial communities in Mh and Mhg, respectively. At the bacterial genus level (Fig. 4B), distinct dominant bacterial genera were identified between the two *Monotropastrum* species. In Mh, the bacterial community was dominated by *Serratia*, *Burkholderia-Caballeronia-Paraburkholderia*, *Bradyrhizobium*, *Cedecea*, and *Klebsiella*. In contrast, the Mhg rhizosphere was primarily dominated by *Serratia*, *Burkholderia-Caballeronia-Paraburkholderia*, *Bradyrhizobium*, *Acidothermus*, and *Pseudomonas*. Consequently, *Burkholderia-Caballeronia-Paraburkholderia*, *Bradyrhizobium*, and *Serratia* were identified as the shared core dominant genera in the rhizosphere soils of both *Monotropastrum* species.

At the fungal phylum level (Fig. 4C), Ascomycota, Basidiomycota and Mortierellomycota represented the dominant fungal communities in both species. In Mh, Basidiomycota was the most abundant phylum (49.57%), followed by Ascomycota (34.81%). Conversely, the Mhg rhizosphere exhibited a higher relative abundance of Ascomycota (48.93%), with Basidiomycota as the second most abundant group (24.24%). At the fungal genus level (Fig. 4D), *Saitozyma* and *Podila* were identified as the shared core dominant genera in both species. Mh showed higher relative abundances of *Russula*, whereas *Lactarius* was distinctively enriched in Mhg rhizosphere.

LEfSe was used to identify taxa with significantly different abundances between the two *Monotropastrum* species (Fig. 4E and 4F). For the bacterial communities, the Mh rhizosphere was significantly enriched in *Cedecea* and *Klebsiella*, whereas the Mhg was characterized by high abundances of *Acidothermus* and g_norank_f_Acetobacteraceae. In terms of fungal communities, *Russula* and *Tricholoma* were significantly enriched in Mh, while *Lactarius* was significantly enriched in Mhg.

### Co-occurrence networks of rhizosphere microbial communities in of two *Monotropastrum* species

To elucidate the complex interactions within the rhizosphere microbial communities of the two *Monotropastrum* species, co-occurrence networks were constructed for both bacterial and fungal communities based on the top 50 OTUs by relative abundance (Fig. 5A). In bacterial networks, Mh and Mhg had the same diameter, while the Mh network exhibited relatively lower density and a shorter average path length (APL) than of the Mhg network. Similarly, the fungal network of Mh was more compact, with smaller diameter and shorter APL compared with Mhg. Positive correlations predominated in all four networks (Fig. 5B), whereas a relatively higher proportion of negative correlations occurred within the Mhg fungal network, indicating more antagonistic relationships. In addition, bacterial networks had more nodes and edges than fungal networks (Fig. 5C). Furthermore, the distribution of the average degree (AD) were further compared between Mh and Mhg using the Wilcoxon rank-sum test (Fig. 5D). No significant difference was detected between the two bacterial networks (*p* = 0.6349), whereas the fungal network of Mh showed a significantly higher AD than that of Mhg (*p* = 0.0298). Collectively, these results suggest that while some differences in network topology were observed descriptively, only the variation in fungal network connectivity (AD) between the two species was statistically supported.

### Functional prediction of microbial communities in the rhizosphere soil of two *Monotropastrum* species

Based on the Functional Annotation of Prokaryotic Taxa (FAPROTAX) database, five major bacterial metabolic pathways with high relative abundances were identified in the rhizosphere soils of both *Monotropastrum* species. The two most dominant functional categories were chemoheterotrophy and aerobic chemoheterotrophy (Fig. 6A). The Wilcoxon rank-sum test revealed that nine bacterial metabolic pathways that differed significantly between two species (*p* < 0.05; Fig. 6B). Among these, nitrogen respiration and nitrate reduction demonstrated notably high relative abundances. Furthermore, several metabolic pathways were unique to the rhizobacterial community of Mh, including plant pathogen and nitrate ammonification pathways.

According to FUNGuild-based functional predictions (Fig. 6C), thirteen major fungal trophic or ecological guilds were identified in the rhizosphere fungal communities. The function with the highest relative abundances included Undefined Saprotroph, Endophyte-Litter Saprotroph-Soil Saprotroph-Undefined Saprotroph, Ectomycorrhizal, and Fungal Parasite-Undefined Saprotroph.

### Relationship between soil rhizosphere microbial community structure and soil physicochemical properties

At the genus level, redundancy analysis (RDA) was performed to assess the relationships between rhizosphere microbial community structures of both species and soil physicochemical properties. For bacterial communities, RDA1 and RDA2 explained 30.57% and 13.70% of the total variation, respectively (Fig. 7A). Among the measured parameters, pH (R^2^ = 0.614, *p* = 0.008), AP (R^2^ = 0.325, *p* = 0.048), SOM (R^2^ = 0.687, *p* = 0.008), and AK (R^2^ = 0.396, *p* = 0.031) were identified as the key factors driving the bacterial community structure. For fungal communities, RDA1 and RDA2 accounted for 18.42% and 16.31% of the total structural variance, respectively (Fig. 7B). pH (R^2^ = 0.451, *p* = 0.01), AN (R^2^ = 0.366, *p* = 0.035), and SOM (R^2^ = 0.369, *p* = 0.021) emerged as the primary factors influencing fungal community composition.

Spearman’s correlation analysis between the top ten dominant bacterial genera and soil physicochemical properties revealed that pH, AN, and TN were significant environmental factors influencing bacterial community distribution (Fig. 7C). AK showed a positive correlation with the abundance of *Bradyrhizobium*, but negative correlations with *Serratia*, *Cedecea*, and *Klebsiella*. Specifically, pH exhibited a significant negative correlation with the relative abundances of *Bradyrhizobium* and *Burkholderia-Caballeronia-Paraburkholderia* (*p* < 0.05). In contrast, both TN and AN showed significant positive correlations with the relative abundances of *Acidothermus* and *Roseiarcus* (*p* < 0.05).

Similarly, pH and AP were identified as the main environmental factors shaping the rhizosphere fungal community composition in both *Monotropastrum* species (Fig. 7D). Specifically, pH exhibited a significant positive correlation with *Trichoderma* (*p* < 0.05), while it showed significant negative correlations with *Saitozyma*, *Podila*, and *Tricholoma* (*p* < 0.05). AP was significantly positively correlated with *Tricholoma* but negatively correlated with *Saitozyma* (*p* < 0.05). In addition, AN was significantly negatively correlated with *Russula* (*p* < 0.05).

## Discussion

### Overall characteristics of the rhizosphere soil microbial community

This study compared the composition of the rhizosphere microbial communities between two closely related *Monotropastrum* species Mh and Mhg. The consistently higher bacterial diversity compared to fungal communities suggests that bacteria may play a more flexible and functionally diverse role in rhizosphere processes, whereas fungal communities in mycoheterotrophic systems are likely constrained by host specificity and symbiotic requirements. This pattern has been reported in other mycoheterotrophic plants, such as *Gastrodia elata* (Chen et al., 2025), and is consistent with the generally higher metabolic plasticity and ecological adaptability of bacterial communities compared to fungi (Cheng et al., 2024). Such a pattern aligns with the ecological characteristics of obligate mycoheterotrophic plants, where fungal communities are closely linked to host-associated symbiotic relationships, while bacterial communities remain more responsive to environmental heterogeneity (Niu et al., 2025).

NMDS analysis further indicated stronger host-associated differentiation in fungal communities than in bacterial communities. Consistent with this pattern, the relatively high proportion of fungal variation explained by plant species suggests strong host-associated differentiation between the two *Monotropastrum* taxa. Such a pattern is biologically plausible because mycoheterotrophic plants depend on specific fungal partners for carbon acquisition, and previous studies have shown that *Monotropastrum* species are closely associated with *Russula*ceae fungi, with Mh and Mhg exhibiting distinct preferences for *Russula* and *Lactarius*, respectively (Liu et al., 2024; Matsuda et al., 2011).

Soil properties also contributed to microbial assembly, likely through their effects on nutrient availability and rhizosphere microhabitat conditions, which is consistent with previous studies highlighting the importance of edaphic factors, especially pH, in shaping rhizosphere microbial communities (Li et al., 2022; Liu et al., 2023). However, because soil variables were analyzed individually, their combined effects were not explicitly evaluated in the present study. Taken together, these findings suggest that rhizosphere microbial assembly in *Monotropastrum* reflects the combined influence host-associated processes and environmental influences, with particularly strong host-related differentiation in fungal communities.

### Dominant microbial communities in the rhizosphere and their interactions with the host plant

Both *Monotropastrum* taxa harbored a conserved core microbiome, suggesting the presence of a functionally stable rhizosphere system. The bacterial community was dominated by Proteobacteria, Acidobacteria, and Actinobacteria, phyla widely recognized for their roles in organic matter decomposition and nutrient cycling (Kalam et al., 2020; Olanrewaju and Babalola, 2019; Timofeeva et al., 2023; Usman et al., 2025). Representative genera within these groups are known to enhance plant stress resistance and nitrogen use efficiency (Kulkova et al., 2024; Pang et al., 2023), further supporting their ecological importance in rhizosphere functioning.

Fungal communities were mainly composed of Ascomycota and Basidiomycota, which are widely distributed root-associated taxa and key decomposers in soil ecosystems (Berlemont et al., 2014; Egidi et al., 2019; Manici et al., 2024). Common dominant genera associated with organic matter decomposition further reinforce the role of these communities in nutrient turnover (Aliyu et al., 2021; Yang et al., 2024). Functional predictions corroborate these ecological roles, suggesting that these rhizosphere microbial communities facilitate the nutrient acquisition by promoting organic matter decomposition and nutrient transformation, particularly nitrogen and phosphorus cycling.

Beyond the shared core microbiome, LEfSe analysis revealed distinct taxa specifically enriched in rhizospheres. The enrichment of the fungal genera *Russula* in the rhizosphere of Mh and *Lactarius* in Mhg corroborates findings from root symbiosis studies, which identified *Russula* as the primary symbiotic partner of Mh roots and *Lactarius* for Mhg roots (Liu et al., 2024; Matsuda et al., 2011). This may reflect a host-mediated recruitment process in which the host selectively attracts its specific fungal partner, reflected externally as significant enrichment of that fungus in the rhizosphere.

Each host also possessed unique bacterial enrichments that appear functionally complementary to its fungal partner. For example, *Acidothermus*, noted for its strong cellulolytic and xylanolytic capacities (Kim et al., 2016), was enriched in the Mhg rhizosphere, complementing the limited lignocellulose-degrading ability of *Lactarius* (Miyauchi et al., 2020) and potentially enhancing nutrient transfer efficiency from fungus to host. Conversely, *Klebsiella*, a bacterium capable of nutrient mobilization (Chen et al., 2024), was enriched in the Mh rhizosphere, possibly cooperating with *Russula*’s efficient nutrient-transport system to sustain Mh nutrition. This pattern illustrates how mycoheterotrophic plants, by selecting a core fungal symbiont, may contribute to the assembly of a cross-kingdom consortium whose synergistic interactions optimize their specialized nutrient-acquisition strategy.

The divergent recruitment of microbial partners between these closely related species may be associated with subtle genetic differentiation accumulated during evolution (Tsukaya et al., 2008). Given that root exudates act as key signaling molecules mediating microbial recruitment (Jin et al., 2024), it is possible that genomic differences between the two hosts lead to variation in exudate profiles that attract distinct symbiotic partners. By forming mycorrhizal associations with different fungal genera, the two plants reduce direct competition for fungal resources and achieve niche differentiation at a broader ecological scale. However, this hypothesis requires further experimental validation.

Co-occurrence network analysis further indicated differences in interaction patterns between the two rhizosphere systems. The coexistence of positive and negative correlations in the Mhg fungal network suggests more complex intra-community dynamics, potentially reflecting competition under resource-limited conditions. These findings suggests that, although host selection establishes the overarching microbial framework, intense intra-kingdom competition persists among recruited fungi. Such competition is likely driven by nutrient limitation, as reflected by the lower SOM content in the Mhg rhizosphere. Under these conditions, fungi sharing similar saprotrophic functions may experience niche overlap in decomposing organic matter and acquiring carbon sources, resulting in antagonistic interactions. The dynamic balance between cooperation and competition thus facilitates the stable coexistence of *Lactarius* and *Acidothermus* through functional complementarity. Meanwhile, under nutrient-limited conditions, the host may selectively favor the most efficient decomposers, enhancing overall nutrient mobilization within the rhizosphere to sustain its mycoheterotrophic lifestyle.

### Influence of soil physicochemical properties on rhizosphere microbial community assembly

In addition to host-associated processes, soil physicochemical properties also contributed to shaping rhizosphere microbial communities. Rather than acting independently, these environmental factors likely function as second-tier ecological filters, fine-tuning the microbial community originally structured by host selection.

Among the measured variables, soil pH showed consistent associations with both bacterial and fungal communities. The rhizosphere soils of both species were consistently acidic, which may play an important role in shaping microbial community structure (Liu et al., 2018). Soil pH is widely recognized as a key environmental factor influencing microbial assemblages. In this study, pH likely influenced microbial communities indirectly by regulating nutrient availability and microbial metabolic activity, rather than directly determining the distribution of specific taxa. This interpretation is consistent with previous studies suggesting that environmental factors drive community composition through their effects on resource availability (Cullings and Makhija, 2001).

Nutrient-related factors, including nitrogen, phosphorus, and potassium, were also associated with variations in microbial community composition. Rather than acting independently, these nutrients likely function as an integrated resource system that shapes microbial assemblages by regulating overall nutrient availability. Changes in nutrient status may influence microbial community structure by altering resource competition and metabolic activity, thereby favoring taxa adapted to specific nutrient conditions. Such effects are consistent with previous studies showing that nutrient availability can modulate microbial community composition and functional potential (Harman et al., 2004; Kaneko et al., 2002; Lu et al., 2022).

For these two obligate mycoheterotrophic plants, nitrogen, phosphorus, and potassium may jointly contribute to variation in rhizosphere microbial community composition by influencing nutrient availability in soil. This may be ecologically important because mycoheterotrophic plants depend strongly on fungal-mediated resource transfer and other belowground microbial processes for nutrition (Bidartondo and Bruns, 2005).

Taken together, these findings indicate that rhizosphere microbial assembly in the two *Monotropastrum* taxa was associated with both host identity and selected soil physicochemical properties, and that the association patterns differed between bacterial and fungal communities. This provides an ecological perspective on how mycoheterotrophic plants maintain stable symbiotic systems under heterogeneous soil environments.

## Conclusion

This study systematically compared the rhizosphere microbial communities of two closely related *Monotropastrum* species and identified the key soil physicochemical properties associated with their community assembly. Although the two plants shared several dominant microbial taxa, each species selectively enriched distinct bacterial and fungal groups in its rhizosphere, indicating host-associated differentiation in microbial recruitment. Soil pH, available nitrogen (AN), available potassium (AK), available phosphorus (AP), and soil organic matter (SOM) were significantly associated with rhizosphere microbial community composition, and the association patterns differed between bacterial and fungal communities. These findings demonstrate that the rhizosphere microbiomes of the two *Monotropastrum* species are shaped by the combined influence of host-associated processes and environmental filtering, resulting in distinct microbial assembly patterns under natural conditions, deepen our understanding of plant–microbe interactions in obligate mycoheterotrophic plants, and provide a scientific basis for exploring microbial inoculants, habitat restoration, and potential cultivation of these ecologically specialized species.

## Figures and Tables

**Fig. 1.**
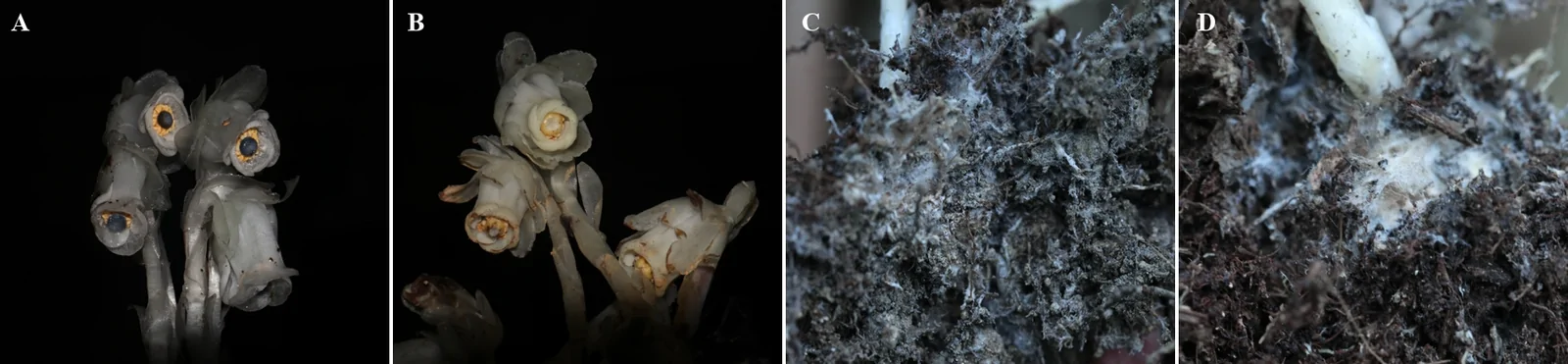
Plant samples of Mh (A) and Mhg (B), mycorrhizal samples of Mh (C) and Mhg (D).

**Fig. 2.**
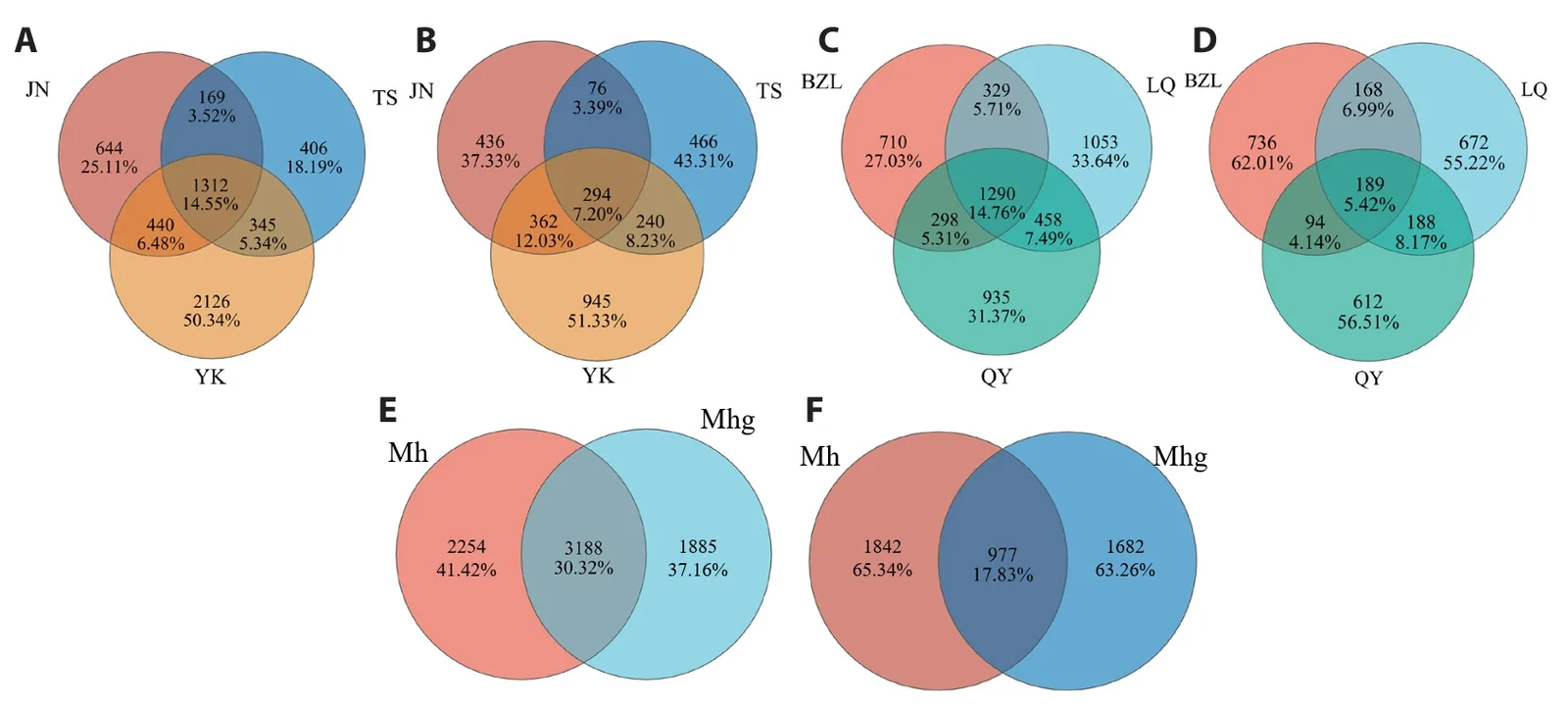
Venn diagram of microbial communities in the rhizosphere soil of two *Monotropastrum* species across different sites. The numbers on each diagram refer to the number of Mh bacterial (A) and fungal (B) rhizosphere soil operational taxon units (OTUs), the number of Mhg bacterial (C) and fungal (D) rhizosphere soil OTUs, the number of bacterial (E) and fungal (F) rhizosphere soil OTUs between two *Monotropastrum* species.

**Fig. 3.**
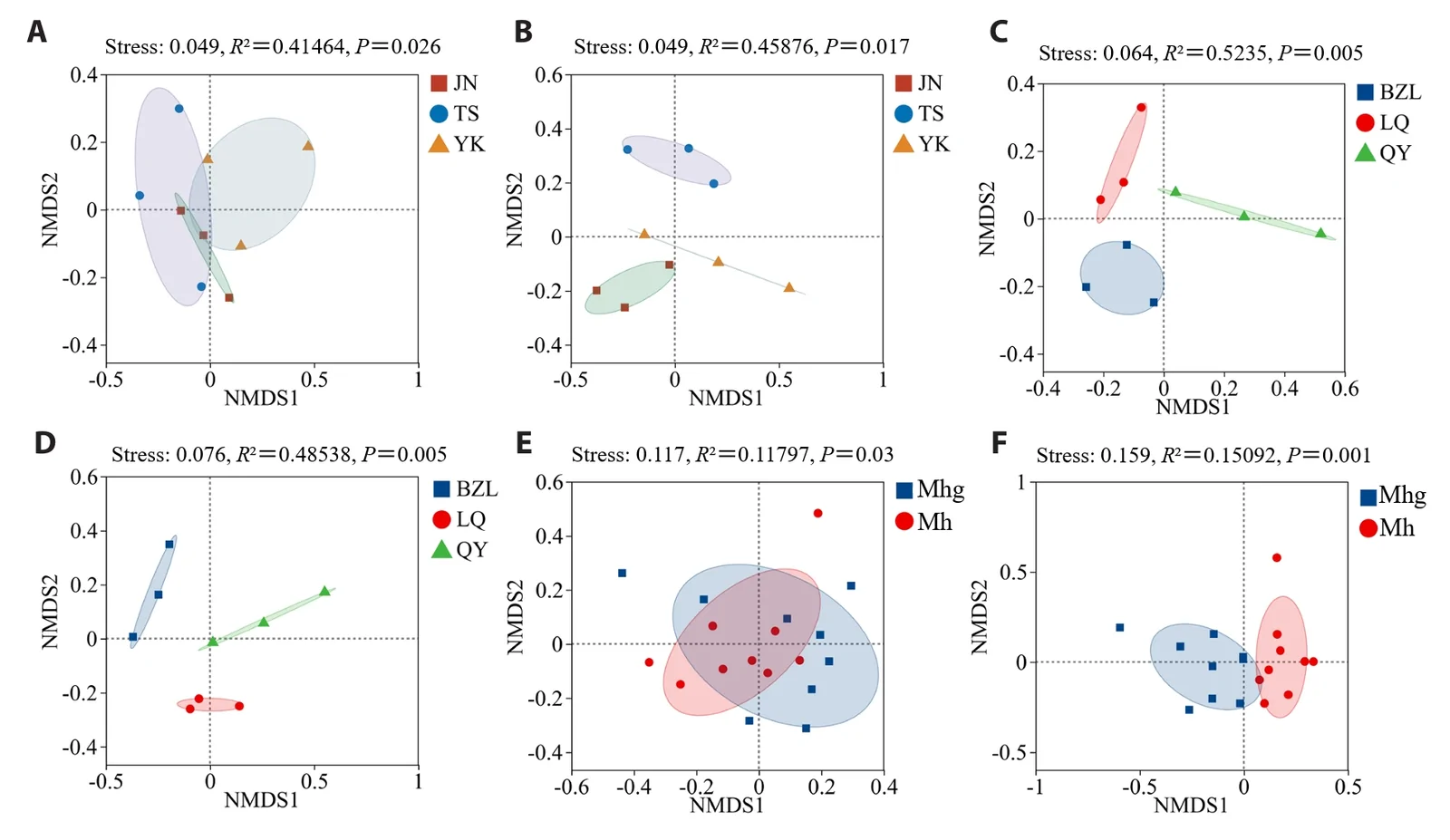
Microbial communities in the soil of two *Monotropastrum* species at various sites were analyzed using non-metric multidimensional scaling (NMDS), based on the Bray-Curtis distance. Distribution of bacterial (A) and fungal (B) communities in the rhizosphere soil of Mh at different sites, distribution of bacterial (C) and fungal (D) communities in the rhizosphere soil of Mhg at different sites, distribution of bacterial (E) and fungal (F) communities in the rhizosphere soil of two *Monotropastrum* species.

**Fig. 4.**
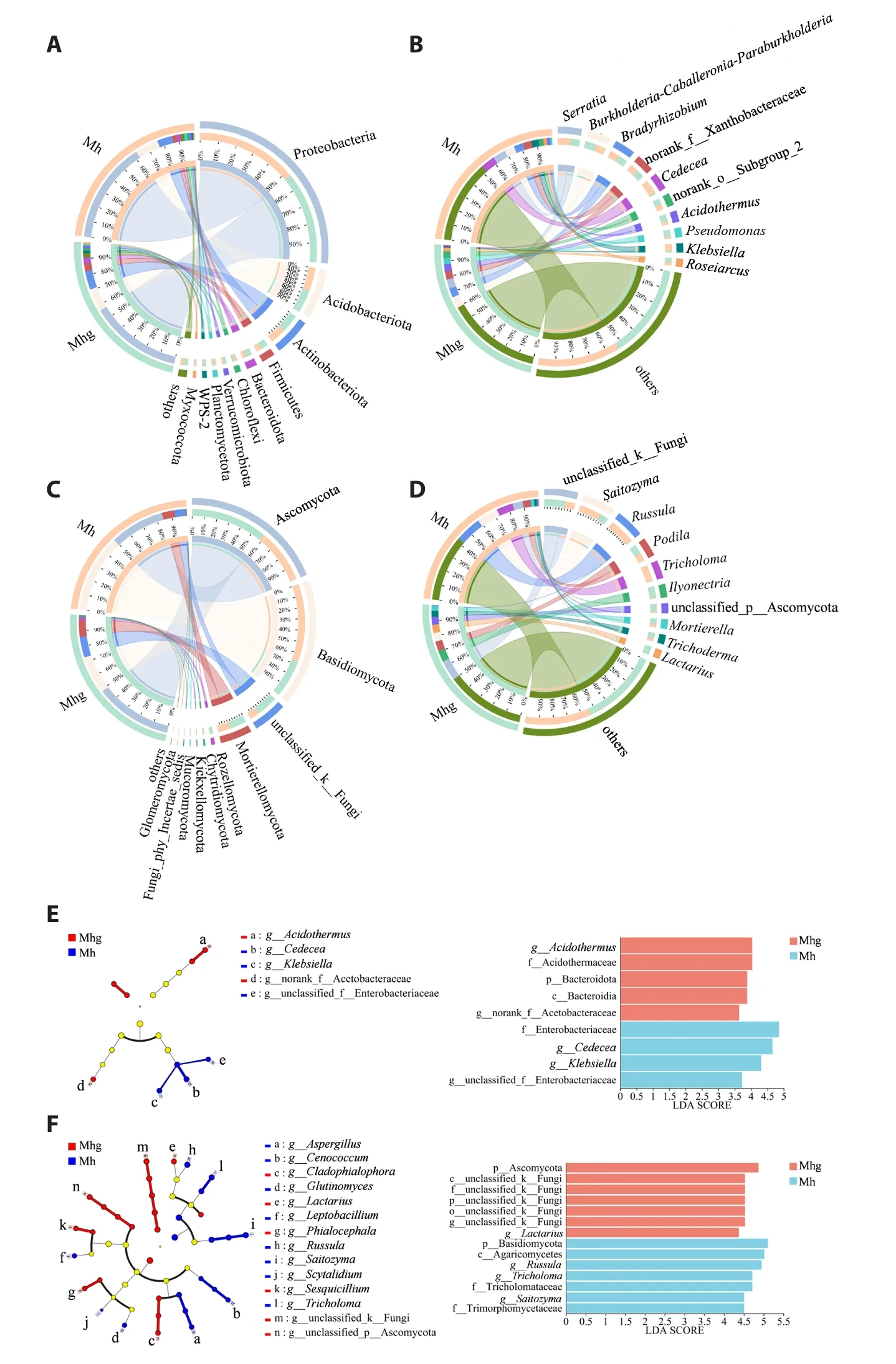
The composition of microbial communities in the rhizosphere soil of two *Monotropastrum* species. Phylum-level (A) and genus level (B) composition of the rhizosphere soil bacterial community in two *Monotropastrum* species, phylum-level (C) and genus level (D) composition of the rhizosphere soil fungal community in two *Monotropastrum* species. Analysis of the microbial communities in the rhizosphere soil of two *Monotropastrum* species using linear discriminant analysis (LDA) effect size (LEfSe) with an LDA threshold of > 3.5. LEfSe analysis of bacteria (E) and fungi (F) in rhizosphere soil between Mh and Mhg.

**Fig. 5.**
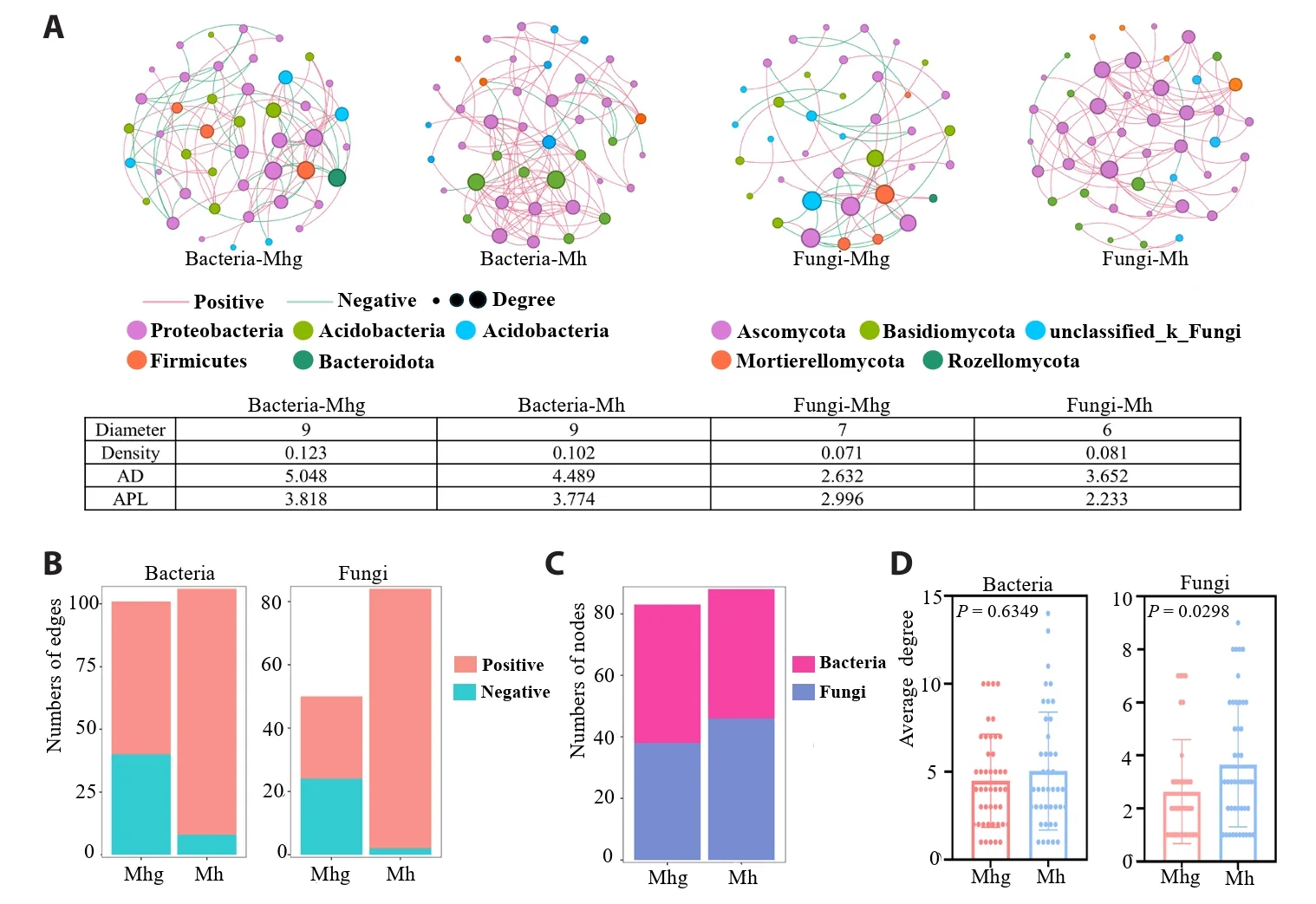
Co-occurrence networks of rhizosphere microbial communities constructed based on the top 50 OTUs ranked by relative abundance. (A) Edges represent significant correlations (Spearman’s correlation coefficient |r| > 0.8, *p* < 0.05); line thickness is proportional to the correlation coefficient and colors indicate positive (green) or negative (red) interactions. Key topological parameters of each network are summarized in the accompanying table. (B) The numbers of edges. (C) The numbers of nodes. (D) Comparison of average degree (AD) between Mh and Mhg networks, differences between Mh and Mhg were tested using the Wilcoxon rank-sum test, with *p* < 0.05 considered statistically significant.

**Fig. 6.**
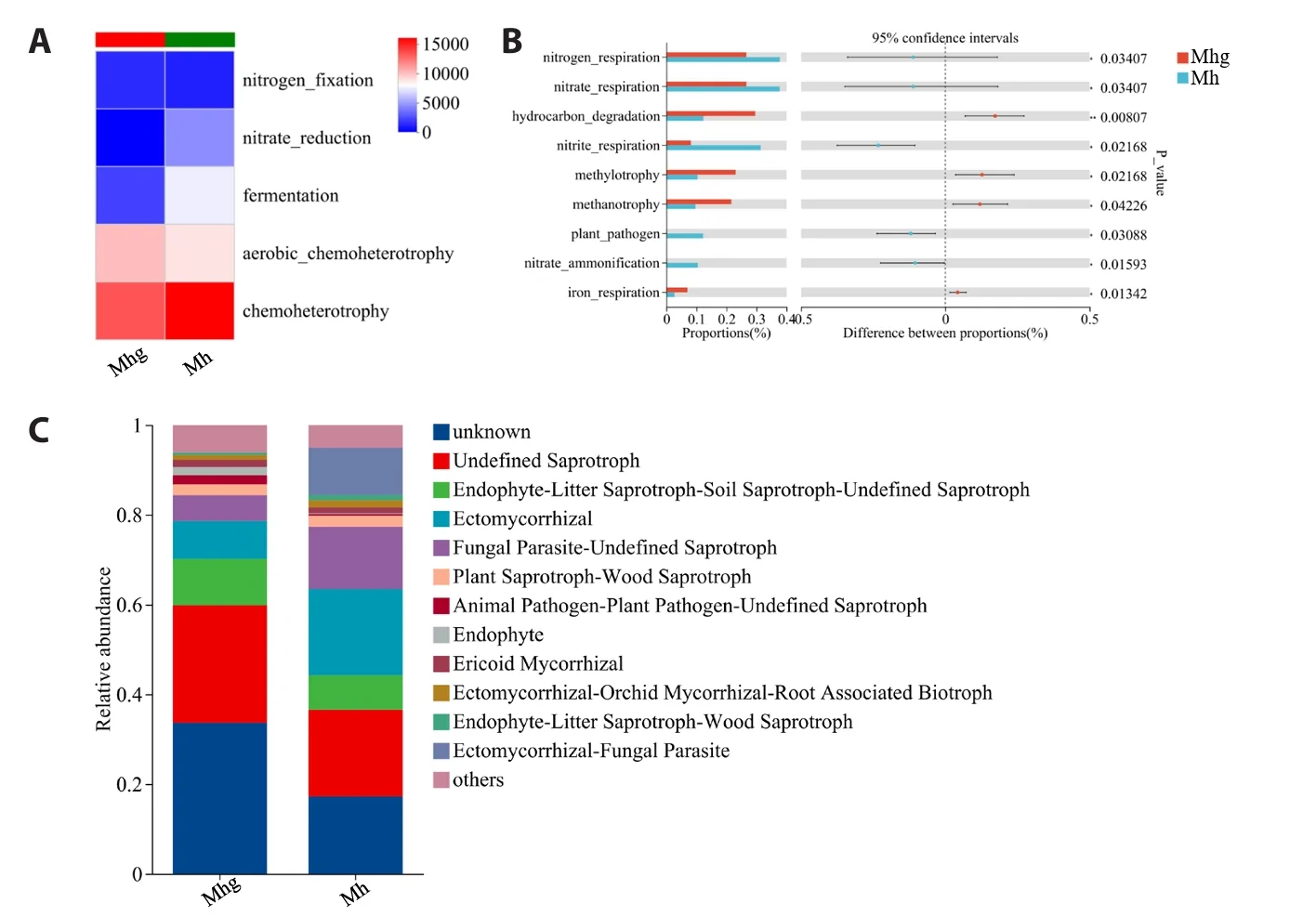
Functional predictions of soil microbial communities in rhizosphere soil of two *Monotropastrum* species. Compositional variability test for bacterial (A, B) and fungal (C) functional groups.

**Fig. 7.**
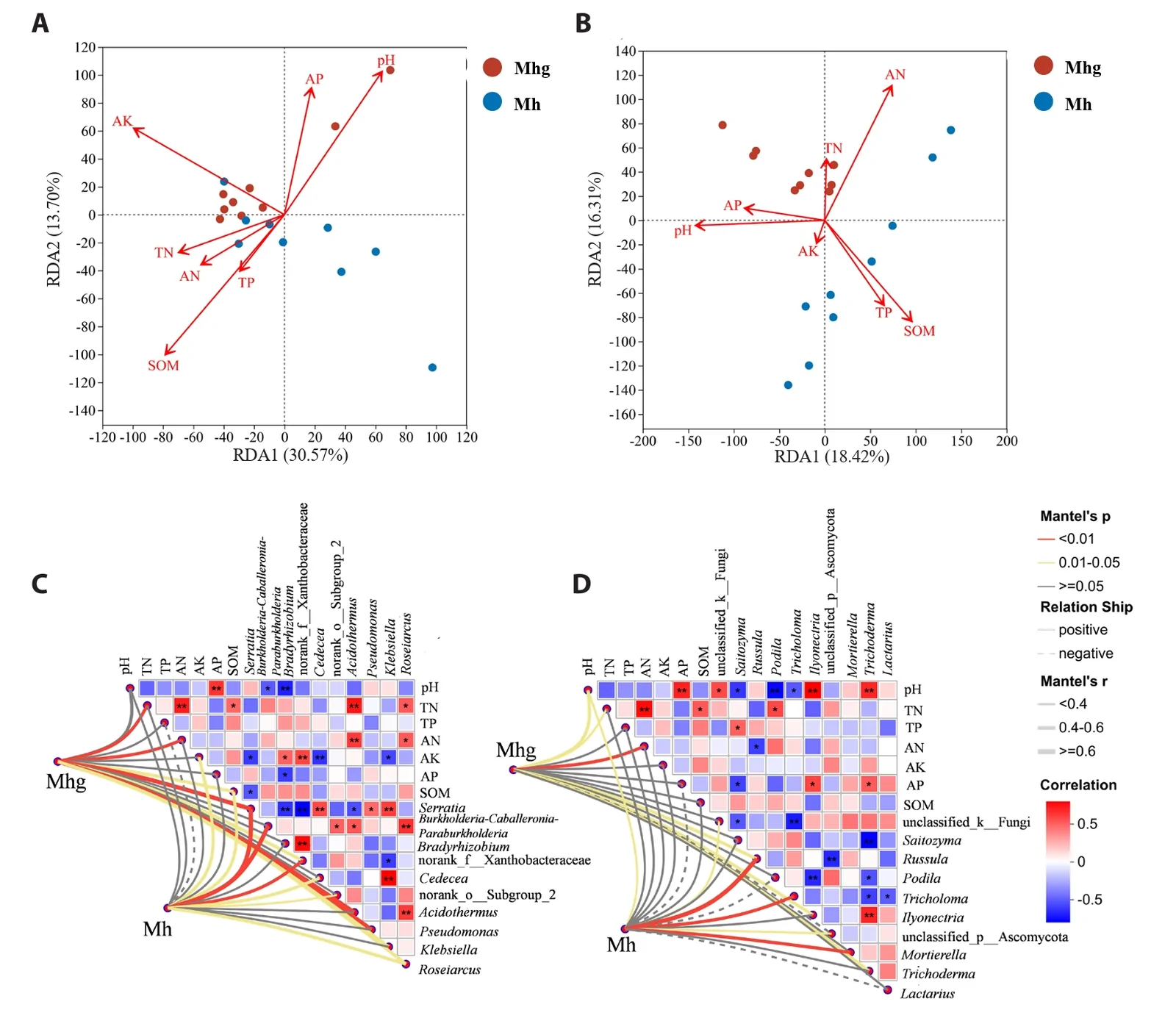
Redundancy analysis (RDA) of (A) bacterial and (B) fungal communities at genus level in relation to soil physicochemical properties. The ordination diagrams illustrate the effects of environmental variables (red line, red arrows) on the sample locations (circles). RDA triplot show correlations between environmental factors (red arrows) and microbial community structure. Spearman correlation heatmaps between soil physicochemical properties and rhizosphere bacterial (C), fungal (D) communities at genus level. “*” represents 0.01 < *p* < 0.05, “**” represents *p* < 0.01, Correlation coefficients range from -1 to 1, where -1 to 0 indicate a negative correlation and 0 to 1 indicate a positive correlation.

**Table 1.** Alpha diversity index of microorganisms in the rhizosphere soil of two *Monotropastrum* species

	Index	Mh			Mhg		
		Jingning (JN)	Taishun (TS)	Yongkang (YK)	Baizhangling (BZL)	Longquan (LQ)	Qingyuan (QY)
Bacteria	Shannon	5.25 ± 0.19a	4.44 ± 0.84a	5.80 ± 0.43a	5.46 ± 0.34a	5.55 ± 0.16a	5.29 ± 0.46a
	Simpson	0.02 ± 0.00a	0.07 ± 0.05a	0.02 ± 0.01a	0.01 ± 0.00a	0.02 ± 0.00a	0.03 ± 0.01a
	ACE	2,000.95 ± 171.27a	1,594 ± 362.93a	3,036.13 ± 416.79b	1,805.28 ± 377.83a	2,247.51 ± 339.72a	2,225.96 ± 216.73a
	Chao1	1,924.25 ± 140.63a	1,541.35 ± 350.22a	2,870 ± 362.49b	1,735.29 ± 348.83a	2,149.85 ± 310.47a	2,155.10 ± 189.87a
Fungi	Shannon	3.37 ± 0.36a	3.34 ± 0.31a	3.99 ± 0.24a	3.63 ± 0.41a	3.90 ± 0.17a	3.99 ± 0.38a
	Simpson	0.13 ± 0.05a	0.11 ± 0.04a	0.10 ± 0.04a	0.07 ± 0.03a	0.06 ± 0.01a	0.06 ± 0.03a
	ACE	686.71 ± 21.15a	532.77 ± 43.31b	1,020.75 ± 40.31c	622.02 ± 151.27a	735.18 ± 50.61a	544.48 ± 26.56a
	Chao1	676.75 ± 22.25a	530.13 ± 36.43b	1,019.16 ± 46.05c	613.41 ± 146.24a	724.19 ± 61.70a	549.50 ± 29.64a

**Table 2.** Comparison of Alpha diversity index of microorganisms in the rhizosphere soil between Mh and Mhg

	Sample	Shannon index	Simpson index	ACE index	Chao1 index
Bacteria	Mh	5.16 ± 0.79a	0.04 ± 0.04a	2,092.92 ± 692.73a	2,112.14 ± 635.22a
	Mhg	5.43 ± 0.36a	0.04 ± 0.01a	2,092.92 ± 378.36a	2,013.42 ± 351.27a
Fungi	Mh	3.57 ± 0.43a	0.12 ± 0.05b	746.74 ± 206.89a	742.02 ± 208.09a
	Mhg	3.84 ± 0.37a	0.06 ± 0.03a	633.89 ± 121.86a	629.03 ± 117.89a

**Table 3.** Multifactor PERMANOVA of the effects of plant species and soil environmental factors on rhizosphere microbial communities

Microorganisms	Characteristics	Df	SumsOfSqs	MeanSqs	F_Model	R²	Pr (> F)
Bacteria	Mh & Mhg	1	0.32146	0.32146	3.27311	0.11797	0.001
	pH	1	0.39263	0.39263	3.99777	0.14409	0.001
	TN	1	0.15019	0.15019	1.52928	0.05512	0.151
	TP	1	0.12342	0.12342	1.25662	0.04529	0.254
	AN	1	0.14033	0.14033	1.42882	0.0515	0.155
	AK	1	0.43134	0.43134	4.39198	0.1583	0.001
	AP	1	0.13347	0.13347	1.359	0.04898	0.198
	SOM	1	0.14819	0.14819	1.50887	0.05438	0.135
	Residuals	9	0.88391	0.09821	0	0.32438	0
	Total	17	2.72494	0	0	1	0
Fungi	Mh & Mhg	5	3.04544	0.60909	3.89335	0.5524	0.001
	pH	1	0.2559	0.2559	1.63574	0.04642	0.05
	TN	1	0.25522	0.25522	1.63137	0.04629	0.049
	TP	1	0.19755	0.19755	1.26277	0.03583	0.222
	AN	1	0.24658	0.24658	1.57617	0.04473	0.058
	AK	1	0.2804	0.2804	1.79236	0.05086	0.015
	AP	1	0.19198	0.19198	1.22714	0.03482	0.248
	SOM	1	0.25785	0.25785	1.64819	0.04677	0.044
	Residuals	5	0.78222	0.15644	0	0.14188	0
	Total	17	5.51313	0	0	1	0

## Supplementary Information

Table S1. Sample collection information

Table S2. Soil physicochemical properties of Mh and Mhg at different sites

Table S3. Comparison of the physicochemical properties of soil between Mh and Mhg
