# Supplementary material for: Rhizosphere microbiome differentiation and soil environmental drivers in two Monotropastrum species
Source: J Microbiol. 2026 Jul 31;64(7):e2602009. doi: 10.71150/jm.2602009 (PMC13687994; doi:10.71150/jm.2602009)
Supplement: Supplementary file 1 [file jm-2602009-Supplementary-Table-S1.pdf]

Table S1. Sample collection information

Samples	Species	Locations	Altitude (m)	Latitude	Longitude
Jingning (JN)	Mh	Dongkeng Town, Jingning County	700.25	119.68	27.78
Taishun (TS)	Mh	Siqian She Ethnic Town, Taishun County	1,036.37	119.48	27.70
Yongkang (YK)	Mh	Zhoushan Town, Yongkang City	276.23	120.24	28.86
Baizhangling (BZL)	Mhg	Qingliangfeng Town, Lin'an City	1,333.04	119.00	30.20
Longquan (LQ)	Mhg	Pingnan Town, Longquan City	1,459.08	119.17	27.89
Qingyuan (QY)	Mhg	Baishanzu Town, Qingyuan County	1,208.01	119.19	27.73
