# Supplementary material for: Rhizosphere microbiome differentiation and soil environmental drivers in two Monotropastrum species
Source: J Microbiol. 2026 Jul 31;64(7):e2602009. doi: 10.71150/jm.2602009 (PMC13687994; doi:10.71150/jm.2602009)
Supplement: Supplementary file 2 [file jm-2602009-Supplementary-Table-S2.pdf]

Table S2. Soil physicochemical properties of Mh and Mhg at different sites

Physicochemical properties	Mh			Mhg		
	Jingning (JN)	Taishun (TS)	Yongkang (YK)	Baizhangling (BZL)	Longquan (LQ)	Qingyuan (QY)
pH	4.80 ± 0.06a	4.98 ± 0.14a	4.98 ± 0.18a	4.99 ± 0.04ab	4.71 ± 0.10b	5.28 ± 0.23a
TN (%)	0.12 ± 0.01a	0.15 ± 0.05a	0.10 ± 0.01a	0.19 ± 0.07ab	0.27 ± 0.00a	0.08 ± 0.03b
TP (%)	0.11 ± 0.00a	0.13 ± 0.01a	0.13 ± 0.01a	0.11 ± 0.01a	0.13 ± 0.02a	0.11 ± 0.00a
SOM (%)	3.30 ± 0.03a	3.29 ± 0.08a	3.46 ± 0.25a	3.25 ± 0.11a	3.39 ± 0.11a	2.16 ± 1.19a
AN (mg/kg)	63.39 ± 16.94a	47.67 ± 5.41a	13.70 ± 5.38b	67.76 ± 7.01a	63.41 ± 19.76a	32.38 ± 13.61b
AP (mg/kg)	10.91 ± 0.61a	10.71 ± 0.35a	11.00 ± 0.96a	11.67 ± 0.67a	9.87 ± 0.34a	11.46 ± 0.54a
AK (mg/kg)	181.58 ± 55.54b	101.97 ± 34.79b	373.49 ± 85.73a	211.21 ± 13.80ab	298.51 ± 35.52a	199.38 ± 37.23b

Note: Data in the table are presented as the Mean ± SD. Each group of values is followed by different lowercase letters to indicate $p < 0.05$. SOM, soil organic matter; TN, soil total nitrogen; AN, alkali-hydrolyzable nitrogen; TP, total phosphorus; AP, available phosphorus; AK, available potassium. The same below.
