# Supplementary material for: Rhizosphere microbiome differentiation and soil environmental drivers in two Monotropastrum species
Source: J Microbiol. 2026 Jul 31;64(7):e2602009. doi: 10.71150/jm.2602009 (PMC13687994; doi:10.71150/jm.2602009)
Supplement: Supplementary file 3 [file jm-2602009-Supplementary-Table-S3.pdf]

Table S3. Comparison of the physicochemical properties of soil between Mh and Mhg

Physical and chemical properties	Mh	Mhg
pH	4.92 ± 0.16a	4.99 ± 0.27a
TN (%)	0.12 ± 0.04a	0.18 ± 0.09a
TP (%)	0.12 ± 0.01a	0.12 ± 0.02a
SOM (%)	3.35 ± 0.17a	2.93 ± 0.89a
AN (mg/kg)	41.59 ± 23.35a	54.52 ± 21.37a
AP (mg/kg)	10.87 ± 0.70a	11.00 ± 0.97a
AK (mg/kg)	219.01 ± 129.88a	236.37 ± 53.86a
